# Schrödinger’s fetus examined

**DOI:** 10.1007/s11019-019-09936-0

**Published:** 2019-12-13

**Authors:** Bruce P. Blackshaw

**Affiliations:** Department of Philosophy, University of Birmingham, Birmingham, UK

**Keywords:** Abortion, Fetus, Person, Actual future principle, Future like ours

## Abstract

Joona Räsänen has proposed a concept he calls Schrödinger’s Fetus as a solution to reconciling what he believes are two widely held but contradictory intuitions. I show that Elizabeth Harman’s Actual Future Principle, upon which Schrödinger’s Fetus is based, uses a more convincing account of personhood. I also argue that both Räsänen and Harman, by embracing animalism, weaken their arguments by allowing Don Marquis’ ‘future like ours’ argument for the immorality of abortion into the frame.

## Introduction

Joona Räsänen has recently proposed a concept he calls Schrödinger’s Fetus (2019) to further develop and defend Elizabeth Harman’s Actual Future Principle (which I will refer to as the AFP) (1999). The AFP states that the moral status of an early fetus is dependent on its actual future: ‘an early fetus that will become a person has some moral status. An early fetus that will die while it is still an early fetus has no moral status’ (Harman 1999).

Given that the actual future of a given early fetus is uncertain, Räsänen has cleverly captured the indeterminacy of the early fetus’ moral status under the AFP with Schrödinger’s Fetus, based on Schrödinger’s cat—a quantum mechanics thought experiment that involves a cat that is simultaneously alive and dead until the state of a radioactive atom is observed. Schrödinger’s Fetus similarly has an indeterminate moral status—whether the fetus is a person with accompanying moral status cannot be known until the fetus has (or has not) gained consciousness.

Harman’s original paper described two contradictory intuitions that the AFP is designed to reconcile: that early fetuses that die in early abortions lack moral status, and that early fetuses that survive to become persons have some moral status. According to Räsänen Schrödinger’s Fetus is designed for two different contradictory intuitions: first, that ‘I was once an early fetus’, and second, that ‘early abortion does not kill anybody, but prevents someone from coming into existence’ (Räsänen 2019).

Here, I show that Harman’s account of personhood is more convincing than Räsänen’s account, but given this is the only substantial difference between the arguments, leaves Schrödinger’s Fetus as offering little more than the AFP. I also show both Räsänen’s and Harman’s embrace of animalism as an account of personal identity opens the door for Don Marquis’ ‘future like ours’ argument against the permissibility of abortion (1989), substantially weakening their case.

## Räsänen’s concept of persons

It is important to note that Räsänen’s argument is subtly different to Harman’s AFP. Under the AFP, a fetus that will one day *become* a person has *some* moral status—it deserves some moral consideration because of certain intrinsic properties it will possess in the future.

Schrödinger’s Fetus, however, *is* a person—as a *fetus*—*if* it will go on to become conscious. According to Räsänen, we must wait until the fetus has reached consciousness to determine if it was always a person, implying that he believes consciousness is crucial to moral status. However, Räsänen seems to be saying that the property of being conscious *at some point in the future* is a sufficient criterion for personhood, and this seems problematic. Essentially, it is a claim that the (certain) *potential* for consciousness confers full moral status. It is unclear how Räsänen can ground this view—it is susceptible to the criticism that he is treating a potential X as if it were an actual X without justification. This is reminiscent of the *substance view of persons,* which is an account of personhood that regards human beings as intrinsically valuable because they are rational moral agents *by nature* (Beckwith 2004). Of course, the substance view cannot be utilised by Räsänen, as *all* early fetuses are rational moral persons by nature and considered persons, contrary to Schrödinger’s Fetus.

Harman’s approach of assigning *some* moral value to a fetus that becomes a person (whatever the criteria) in the future seems far easier to justify than Räsänen’s account, and yet without his claim regarding personhood of Schrödinger’s Fetus, his account is merely the AFP rebadged.

## Räsänen’s intuitions

Räsänen designed Schrödinger’s Fetus to deal with two contradictory intuitions that he claims are widely shared. The first is that ‘I was once an early fetus’ and it seems self-evident that this intuition is broadly accepted. Indeed, it is the basis for the account of personal identity known as animalism—a view that claims we are animals, and as long as our animal functions survive, we survive. Räsänen confirms that he is referring to animalism by citing the *Too Many Thinkers* problem, an important defence of animalism (Hershenov 2013). On animalism, we come into existence at or soon after conception.^1^ Elizabeth Harman holds a similar view, stating that ‘what we are is biological living organisms, with the same criteria of identity we would apply to other animals’ (1999).

Räsänen’s second intuition is ‘early abortion does not kill anybody, but prevents someone from coming into existence’ (2019). Contra Räsänen, I am doubtful this is a widely held intuition. It certainly contradicts the intuition that I was once an early fetus (of course Räsänen agrees, and proposes Schrödinger’s Fetus as a solution), but it also seems unlikely that a pregnant woman who miscarries consoles herself by reasoning that her miscarriage did not involve the death of anyone but merely preventing someone from coming into existence.

One difficulty with Räsänen’s second intuition is that if animalism is accepted—and he certainly seems to accept it—early abortion *always* kills someone, if by ‘someone’ we mean an individual human organism. On animalism, we have *already* come into existence by the time of early abortion—there is no uncertainty as to the status of our existence. Räsänen has confused personal identity with personhood here—Schrödinger’s Fetus is a person if it goes on to become fully conscious, but in terms of personal identity, it is always *someone*, a distinct human being with an identity, no matter what its future.

I suspect what Räsänen actually means is that ‘early abortion does not kill a *person*, but prevents that person from coming into existence’. If we take this interpretation of ‘someone’, Schrödinger’s Fetus allows these two intuitions to be consistent, given only fetuses that survive to become conscious are regarded as persons, and early abortion prevents a fetus from ever being a person.

## A future like ours

Further difficulties arise with Räsänen’s reliance on animalism. Indeed, I find it puzzling why Räsänen and Harman embrace such an account of personal identity, especially since it seems to be less widely accepted than psychological accounts of personal identity. They seem to find it a convincing view, but by accepting the intuition that ‘I was once an early fetus’, Don Marquis’ influential ‘future like ours’ argument comes into play (1989).

According to Marquis, abortion is immoral because it deprives a fetus of a future like our future—and to deprive us of our futures is immoral. Whether or not a fetus is a person is irrelevant to Marquis—what is important is that when a fetus is killed, their future (which is a valuable future as eventually they will be a person, like us) is taken away from them. A crucial requirement for Marquis’ argument to be coherent is that it is the *same individual* that persists from an early fetus to adulthood, otherwise the early fetus would not have a future like ours. Of course, this is precisely what the intuition that ‘I was once an early fetus’ is claiming, as is animalism.

Thus, by embracing animalism, Räsänen introduces a severe complication to Schrödinger’s Fetus. Moral status aside, he needs to explain why depriving an early fetus of a valuable future is not immoral. Given that the usual means of doing so is by denying the intuition that ‘I was once an early fetus’ and rejecting animalism, it will be difficult to do so.

Harman is aware of this difficulty, stating in a footnote that ‘I think we must accept that abortion deprives fetuses of possible futures that would be good’ (1999). In her view, because aborted fetuses lack moral status under the AFP, this is of no moral import. Harman fails to appreciate that according to Marquis, abortion is *prima facie* seriously morally wrong *independently* of the moral status of the fetus.

From Räsänen and Harman’s perspective, a far more persuasive approach would be to reject animalism in favour of a psychological account of personal identity, and reject the intuition that I was once a fetus. This would disarm Marquis’ argument, given that it would entail an individual like us does not come into existence until late in the gestation period.

## Conclusion

When we examine Räsänen’s reasoning for Schrödinger’s Fetus, it is wanting. In requiring that the fetus is a person if it becomes conscious, his account is treating the certain potential for consciousness as being the criterion for personhood. This is difficult to justify if consciousness itself is the morally relevant property. Harman’s AFP, which assigns *some* moral status to a fetus that *will* become a person when it has acquired certain intrinsic properties is substantially more coherent. However, if Räsänen adopted this view, Schrödinger’s Fetus is little more than the AFP with a clever name.

Räsänen’s two intuitions that he tries to reconcile via Schrödinger’s Fetus also are problematic. The first intuition, that I was once an early fetus (shared by Harman), introduces a reliance on animalism, while the second, that early abortion kills no-one but prevents someone from coming into existence, does not seem widely supported and conflicts with animalism irrespective of the moral status of the fetus. More importantly, animalism brings Marquis’ ‘future like ours’ argument into the frame, and this is difficult to counter if animalism is accepted.

A more persuasive approach for both Schrödinger’s Fetus and Harman’s AFP would be to deny the intuition that I was once a fetus, and reject animalism for a psychological account of personal identity.

## Footnotes

1 The possibility of twinning in the first 14 days has resulted in debate over whether the same individual persists from conception.
